# A comprehensive review on beneficial dietary phytochemicals in common traditional Southern African leafy vegetables

**DOI:** 10.1002/fsn3.643

**Published:** 2018-04-14

**Authors:** Dharini Sivakumar, Lingyun Chen, Yasmina Sultanbawa

**Affiliations:** ^1^ Phytochemical Food Network Research Group Department of Crop Sciences Tshwane University of Technology Pretoria South Africa; ^2^ Department of Agricultural, Food and Nutritional Science University of Alberta Edmonton AB Canada; ^3^ Centre for Nutrition and Food Sciences, Queensland Alliance for Agriculture and Food Innovation The University of Queensland Coopers Plains Qld Canada

**Keywords:** β‐ carotene, *Amaranthus* spp, Cowpea, Flavonols, glucosinolates, Moringa leaves, Nightshade and Chinese cabbage, phencolic acids

## Abstract

Regular intake of sufficient amounts of certain dietary phytochemicals was proven to reduce the incidence of noncommunicable chronic diseases and certain infectious diseases. In addition, dietary phytochemicals were also reported to reduce the incidence of metabolic disorders such as obesity in children and adults. However, limited information is available, especially on dietary phytochemicals in the commonly available traditional leafy vegetables. Primarily, the review summarizes information on the major phytochemicals and the impact of geographical location, genotype, agronomy practices, postharvest storage, and processing of common traditional leafy vegetables. The review also briefly discusses the bioavailability and accessibility of major phytochemicals, common antinutritive compounds of the selected vegetables, and recently developed traditional leafy vegetable‐based food products for dietary diversification to improve the balanced diet for the consumers. The potential exists for better use of traditional leafy vegetables to sustain food security and to improve the health and well‐being of humans.

## Abstract 2

The review also briefly discusses the bioavailability and accessibility of major phytochemicals, common antinutritive compounds of the selected vegetables, and recently developed traditional leafy vegetable‐based food products for dietary diversification to improve the balanced diet for the consumers.

## 1 INTRODUCTION

Dietary phytochemicals such as phenolic acids and flavonoids are rich in fruits and vegetables (Liu, 2013). These phenolic compounds are known as natural secondary plant metabolites that mainly participate in the defense mechanism in the plants and provide protection against abiotic or biotic stress. The World Health Organization's (WHO) global initiative program recommends the intake of traditional vegetables in sub‐Saharan Africa due to their non‐nutrient bioactive compounds that possess health‐promoting and protective properties (Smith & Eyzaguirre, 2007). As a result, use of traditional vegetables to combat specific nutrient deficiencies and to sustain secure food has been researched intensively in South Africa (Lewu & Mavengahama, 2010). A set of 21 indigenous leafy vegetable‐based foods from 12 species of indigenous vegetables are currently included in the South African food composition database (Bvenura & Afolayan, 2015). Some traditional vegetables, *Vernonia amygdalina* (bitter leaf)*, Solanum africana, Amaranthus hybridus* (green tete)*,* and *Telfaria occidentalis* (fluted pumpkins), possess higher protein content for dietary applications. Leaves of traditional vegetables from Botswana, *Momordica balsamina* and *Vigna unguiculata subsp* sesquipedalis, exhibit a higher mineral content (K, Fe, Mn, Zn) (Flyman & Afolayan, 2008). Amaranthus spp (Morogo), *A*. *dubius, A. gangetica, A. hybridus, A. spinosus*,* Cucumis metuliferus* (African horned cucumber)*, Cleome monophylla* (Mujakari)*, Ceratotheca triloba* (Wild foxglove)*, Galinsoga parviflora (*potato weed), *Justicia flava* (Yellow justicia), *Momordica balsamina* (nkaka)*, Physalis viscosa* (groundcherry)*,* and *Wahlenbergia undulata* (Cape bluebell), provide mineral concentrations (Ca, P, Na, Zn, Mg, Mn, and Fe) that are higher than the commonly consumed commercial vegetables (Odhav, Beekrum, Akula, & Baijnath, 2007). Apart from the above, traditional African leafy vegetables are rich sources of antioxidants (Uusiku, Oelofse, Duodu, Bester, & Faber, 2010) and provide important dietary phytochemicals to provide protection against noncommunicable disease and obesity (Bohn, Meyer, & Rasmussen, 2008). However, the information on the dietary phytochemical composition in traditional leafy vegetables consumed in the Southern African region is limited (Hervert‐Hernández, García, Rosado, & Goñi, 2011); Moyo et al., 2013). Therefore, the aim of this review was to first summarize the available information primarily on dietary phytochemicals in common traditional leafy vegetables and briefly discuss the influence of geographical location, genotype, agronomy practices, postharvest storage, and processing of phytochemicals. The second aim was to discuss the bioavailability of phytochemicals and the influence of antinutritional components, and finally, the recently developed food products based on traditional leafy vegetables.

## 2 DIETARY PHYTOCHEMICALS IN TRADITIONAL LEAFY VEGETABLES

### 2.1 Phenolic acids and flavonoids

Predominant phenolic acids and flavonols in Amaranthus spp (leaves) are summarized in Table 1. Leaves of different Amaranthus species showed a higher quercetin content than the seed (Kalinova & Dadakova, 2009). Mature leaves of *A. hybrid* (K‐526) exhibited a higher quercetin (15,600 mg/kg Dw) content than *Amaranthus hypochondriacus* (7375 mg/kg Dw), *A. caudatus* (6695 mg/kg Dw) (Kalinova & Dadakova, 2009), and *A. tricolor* (1395 mg/kg Dw) (Kalinova & Dadakova, 2009). Rutin was identified as the predominant quercetin glycoside in Amaranthus species (Kalinova & Dadakova, 2009), and it varied between the different Amaranthus spp, in descending *order*:* A. hybrids* (27,500 mg/kg DW) > *A. hypochondriacus* (13,950 mg/kg DW) ≥ *A. retroflexus* (13,050 mg/kg DW)  > *A. caudatus* (12,010 mg/kg DW)  > *A. tricolor* (2385 mg/kg DW) (Kalinova & Dadakova, 2009). Rutin content in *A. hybrids* and *A. hypochondriacus* is higher than the concentration reported in Elder flower tea (10,900 mg/kg DW) (Cejpek, Malouskova, Konecny, & Velisek, 2009). Furthermore, *A. hybrids* and *A. cruentus* can be recommended as rich sources of rutin and these two Amarathus spp can provide 10–20 kg of rutin/hectare (Kalinova & Dadakova, 2009). Rutin exhibits anti‐inflammatory and anticarcinogenic properties which provide protection against atherosclerosis, osteoarthritis, hemorrhoids, and stroke (Raghav, Gupta, Agrawal, Goswami, & Das, 2006). Myricetin (77 mg/kg) and kaempferol (20 mg/kg) were also reported to be present in *A. hybrids* (leaves) on a fresh weight basis (Medoua & Oldewage‐Theron, 2014).

**Table 1:** Predominant phenolic acids and flavonols, and betalains Amaranthus spp. (leaves)

*Amaranthus* spp (leaves)	Phytochemicals	References
Quercetin (flavonol)		
* A. hybrid* (K‐526)	15,600 mg/kg Dw	Kalinova and Dadakova (2009)
* A. hypochondriacus*	7375 mg/kg Dw	Kalinova and Dadakova (2009)
* A. caudatus*	6695 mg/kg Dw	Kalinova and Dadakova (2009)
Rutin (flavonol)		
* A. hybrids*	27,500 mg/kg DW	Kalinova and Dadakova (2009)
* A. hypochondriacus*	13,950 mg/kg DW	Kalinova and Dadakova (2009)
* A. retroflexus*	13,050 mg/kg DW	Kalinova and Dadakova (2009)
* A. caudatus*	12,010 mg/kg DW	Kalinova and Dadakova (2009)
* A. tricolor*	2385 mg/kg DW	Kalinova and Dadakova (2009)
Myricetin (flavonol)		
* A*. *hybrids*	77 mg/kg FW	Medoua and Oldewage‐Theron (2014)
	Kaempferol	Medoua and Oldewage‐Theron (2014)
	20 mg/kg FW	
Ellagic acid (phenolic acid)		
* A. hypochondriacus*	0.0209 mg/kg FW	Khanam and Oba (2013)
	Gallic acid	
	0.0107 mg/kg	Khanam and Oba (2013)
Betalains		
* A. tricolor*	0.9–1.3759 mg/kg FW	Khanam and Oba (2013)
* A. hypochondriacus*	0.56–0.78 mg/kg FW	Khanam and Oba (2013)

DW, dry weight; FW, fresh weight.

Cultivars of *A. hypochondriacus* (AH6‐1, New Aztec, KANTO 4, Mexico line) contain a higher concentration of hydroxycinnamic acids such as p‐coumaric acid, ferulic acid, sinapic acid, and caffeic acid (0.03–0.059 mg/kg on a fresh weight basis) (Khanam & Oba, 2013). Chlorogenic acid was not detected in *A. hypochondriacu*s cultivars, but it was found in only *A. tricolor* cultivars (Khanam & Oba, 2013). Hydroxybenzoic acid such as gallic acid, vanilic acid, syringic acid, p‐hydroxybenzoic acid, salicylic acid, and ellagic acid is present in *A. hypochondriacus* and *A. tricolor* (Rocto alta, Rocto ranga, Alto pati Baromashi) (Khanam & Oba, 2013). The same authors detected higher ellagic acid (0.0209 mg/kg) and gallic acid (0.0107 mg/kg) on a fresh weight basis in *A. hypochondriacus* cultivars New Aztec and KANTO 4, respectively. Ellagic acids show health benefits due to their antiproliferative and antioxidant properties (Narayanan, Geoffroy, Willingham, Re, & Nixon, 1999; Seeram, Adams, & Henning, 2005). Antidiabetic properties of ferulic acid are well documented (Parmar & Rupasinghe, 2014). Gallic acid was reported to possess anticancer, antioxidant, antimicrobial activities, and cardioprotective effects (Okuda, Yoshida, & Hatano, 1995; Zenebe & Pechanova, 2002). Sinapic acid in *A. hypochondriacus* was higher than *A. tricolor* (Khanam & Oba, 2013). *Amaranthus hypochondriacus,* Mexico line, contains (49.7 mg/kg FW) (Khanam & Oba, 2013) a more or less similar concentration as oranges—tangor (*C. reticulata x C. sinensis*) (50.1 mg/kg DW) (Nićiforović & Abramovič, 2014). Sinapic acid has many health benefits due to their strong antioxidant activity with antimicrobial, anti‐inflammatory, anticancer, and anti‐anxiety activities (Nićiforović & Abramovič, 2014).

Betalains (nitrogen‐containing water‐soluble plant pigments) are further defined betalains as purple betacyanins (purple) (e.g., amaranthin) and betaxanthins (yellow) (Khanam & Oba, 2013). Betalains in *A. tricolor* (0.9–1.3759 mg/kg FW) are higher than in *A. hypochondriacus* (0.56–0.78 mg/kg FW) (Khanam & Oba, 2013) (Table 1). Due to the increased consumer demand for natural food products, betalains have been approved as a natural food additive (coded as E‐162) by the European Union (Khanam & Oba, 2013; Ravichandran et al., 2013). Areal parts of *A. cruentus* and *A. hybridus* contained betacyanin of 40.4 and 6.4 mg amaranthin equivalent/100 g (FW) (Fresh Weight), respectively (Nana, Hilou, Millogo, & Nacoulma, 2012). Betacyanin is associated with numerous health benefits such as cytotoxic (Khan, 2016), neuroprotective (Wang & Yang, 2010), antidiabetic (Lugo‐Radillo, Delgado‐Enciso, & Pena‐Beltrãn, 2012), and hepatoprotective effects (Lee, Wettasinghe, Bolling, Ji, & Parkin, 2005).

*Moringa oleifera,* belonging to the family moringaceae, is another popular traditional vegetable in the Southern African region. It has been identified as a sustainable crop to fight food insecurity (Tshetlhane, 2016). Reports of HPLC analysis of Mexican cultivars (San Pedro and Lombardia) revealed the presence of phenolic acids (gallic and chlorogenic acids) and flavonoids (rutin, luteolin, quercetin, apigenin, and kaempferol) in moringa leaves (Valdez‐Solana et al., 2015). Total phenolic compounds were responsible for the higher antioxidant activity in moringa leaves; they varied from 427.16 to 304.63 μmol Trolox equivalence/g DW (dry weight). Furthermore, ferulic acid was the predominant hydroxycinnamic acid derivative in moringa leaves and the concentration of ferulic acid in moringa leaf is similar to that of orange, eggplant, spinach, red cabbage, and peanuts which is remarkably lower than levels noted in cereals (Leone et al., 2015). The, salicylic acid concentration in *M. oleifera* leaves was similar to the levels found in some commercial fruits and vegetables such as nectarine, pineapple, tomato, and asparagus (Leone et al., 2015). Presence of different isomers of chlorogenic acid [3‐acyl, 4‐acyl, and 5‐acyl p‐coumaroylquinic (pCoQA), caffeoylquinic (CQA), and feruloylquinic acids (FQA), a single isomer of 3,5‐diCQA, 3‐CQA‐glycoside, and two regional i**s**omers of the (3′ and 4′) glycosides of 4‐CQA] was reported in *M. ovalifolia* leaves obtained from Namibia, in the southern region of Africa (Makita et al., 2017). *Moringa oleifera* leaves contain higher levels of myricetin (1296.6 mg/kg DW), quercetin (1362.6 mg/kg DW), and kaempferol (1933.7 mg/kg DW) than commonly consumed spinach (myricetin 620.0 mg/kg DW, quercetin 17.9 mg/kg DW, kaempferol 215.3 mg/kg DW) (Pakade, Cukrowska, & Chimuka, 2013). Cowpea (*Vigna unguiculata*) leaves are also widely consumed and contain quercetin, kaempferol, and isorhamnetin. Among the three flavonols quantified, the content of quercetin was notably higher (Mduma, 2010) (Table 2).

**Table 2:** Flavonoid components in moringa (*Moringa oleifera*), cowpea (*Vigna unguiculata*), and nightshade (*Solanum nigrum*) and Chinese cabbage (*Brassica rapa* sub sp *chinensis*) leaves

Traditional leafy vegetable	Flavonoid component	References
*Moringa* spp		
* Moringa oleifera*	Ferulic acid	
	66.1–96.9 mg/kg DW	Leone et al. (2015)
	Myricetin	
	1296.6 mg/kg DW	Pakade et al. (2013)
	Quercetin	
	1362.6 mg/kg DW	Pakade et al. (2013)
	Kaempferol	
	1933.7 mg/kg DW	Pakade et al. (2013)
* Vigna unguiculata*	Quercetin	
* V. unguiculata* var. Dakawa	654.8 mg/kg DW	Mduma (2010)
* V. unguiculata* var. Ex‐Iseke	585.8 mg/kg DW	Mduma (2010)
* V. unguiculata* var. Dakawa	Kaempferol	
	63.7 mg/kg DW	Mduma (2010)
* V. unguiculata* var. Ex‐Iseke	224.5 mg/kg DW	Mduma (2010)
* V. unguiculata* var. Dakawa	Isohamnetin	
	82.9 mg/kg DW	Mduma (2010)
* V. unguiculata* var. Ex‐Iseke	117.8 mg/kg DW	Mduma (2010)
* Solanum nigrum*	Luteolin	
	1091 mg/kg DW	Huang et al. (2010)
	Apigenin	
	354.06 mg/kg DW	Huang et al. (2010)
	Kaempferol	
	315.65 mg/kg DW	Huang et al. (2010)
* Brassica rapa* sub sp *chinensis*	Quercetin	
	7.44 mg/kg DW	Mampholo et al. (2013)

DW, Dry weight.

HPLC analysis of leaf extract of *Solanum nigrum* exhibited 13 flavonoids that include epigallocatechin, epicatechin, epigallocatechin gallate, gallocatechin gallate, catechin, rutin, naringenin, luteolin, myricetin, quercetin, apigenin, kaempferol, and hesperetin (Huang, Syu, & Lin, 2010). The same authors detected 10 phenolic acids (hydroxybenzoic acid group) such as gallic acid, protocatechuic acid, chlorogenic acid, gentistic acid, vanillic acid, caffeic acid, syringic acid, *p*‐coumaric acid, ferulic acid, and *m*‐coumaric acid. The findings of Huang et al. (2010) confirmed that the leaves of *S. nigrum* are a richer source of phenolic compounds than the stem and fruits. *Brassica rapa* subsp *chinensis* is a nonheading leafy vegetable (Chinese cabbage) and it was reported to contain 7.44 mg/kg quercetin at harvest on a dry weight basis (Mampholo, Sivakumar, Beukes, & Jansen van Rensburg, 2013). The flavonoid components in moringa, cowpea, nightshade, and Chinese cabbage leaves are summarized in Table 2.

### 2.2 Carotenoids

*Amaranthus hybridus* leaf contains 1136 mg/kg total carotenoids and 184 mg/kg β‐carotene Ibrahim et al. (2015), and the composition was comparable to baby spinach (Bergquist, 2006). It is also well known that β‐carotene is a precursor for vitamin A and it shows higher antioxidant properties which provide protection against free radical attack and thereby reducing the incidence of cataracts and cardiovascular disease, enhancing the immune response, and reducing the risk of degenerative diseases such as cancer and muscular, degenerative diseases (Krinsky, 1993). The recommended dietary allowance (RDA) for vitamin A for 19 to 50‐year‐old male and female is 900 μg RAE and 700 μg RAE, respectively (Johnson & Russell, 2010). Also, the fresh leaves of *A. tricolor* and *A. spinosus*. were reported to contain 54.1 mg/kg (Akinyi Nyonje, 2015; Gupta, Lakshmi, Manjunath, & Prakash, 2005) and 53.87 mg/kg β‐carotene (Agea et al., 2014), respectively. Leaves of *Amaranthus* spp were recommended as a good source of vitamin A (Van Jaarsveld et al., 2014), and it meets more than 75% of the RDA. The β‐carotene content in three Amaranthus spp (leaves) is mentioned in Table 1. Furthermore, consumption of leaves of Spider flower, Black nightshade, Tsamma melon, and Jew's mallow fulfills 50%–75% the RDA (Van Jaarsveld et al., 2014).

Spider flower, which includes *Cleome gynandra, C. monophylla* and *C. hirta,* (Capparaceae family), is also a commonly consumed traditional vegetable in the southern African region (Jansen van Rensburg et al., 2007). *Cleome hirta,* a commonly consumed leafy vegetable in Zimbabwe (Codd, Kers, Killick, Tölken, & Marsh, 1970), contains 131.705 mg/kg DW of β‐carotene (Agea et al., 2014). *Corchorus trilocularis* L, known as the member of Jew's mallow (Tiliaceae family) (Jansen van Rensburg et al., 2007), was reported to contain 54.43 mg/kg Dw (Agea et al., 2014). Jew's mallow is commonly consumed by the African communities from Limpopo, Gauteng, and Mpumalanga provinces in South Africa.

Moringa leaves are also recommended as a higher source of β‐carotene, and the content of β‐carotene in moringa leaves was higher than in orange, carrots, and melon, which are known as the primary vegetable sources of β‐carotene (Leone et al., 2015). Cowpea leaves are a rich source of source of β‐carotene, and it contributes to 22.55% of the total carotenoid content and lutein contributes only 4.14% to the total carotenoid content (Mduma, 2010). Furthermore, the total carotenoid content in *Brassica rapa* subsp. *Chinensis, Solanum retroflexum,* and *A. cruentus* was reported as 3.53 mg/kg DW, 268,900 mg/kg DW, and 161,800 g/kg DW at harvest, respectively (Mampholo, Sivakumar, & Jansen Van Rensburg, 2015). Recently, Agea et al. (2014) reported that *S. nigrum* L., commonly known as European black nightshade, contains 131.705 mg/kg FW of β‐carotene. β‐carotene content in selected traditional leafy vegetables is summarized in Table 3.

**Table 3:** β‐carotene composition in different traditional (African) leafy vegetables

Traditional leafy vegetable	β‐carotene	References
*Amaranthus* spp		
* A. hybridus*	184 mg/kg FW	Ibrahim et al. (2015)
* A. tricolor*	54.1 mg/kg FW	Gupta et al. (2005) Akinyi Nyonje (2015)
* A. spinosus*	53.87 mg/kg FW	Gupta et al. (2005) Akinyi Nyonje (2015)
Spider flower		
* Cleome hirta*	131.705 mg/kg DW	Agea et al. (2014)
Jew's mallow		
* Corchorus trilocularis*	54.43 mg/ kg DW	Agea et al. (2014)
*Moringa* spp		
* Moringa* oleifera	100.1 to 285. mg/ kg DW	Agea et al. (2014)
Black nightshade		
* *Solanum *nigrum*	131.705 mg/kg FW	Agea et al. (2014)

DW, dry weight; FW, fresh weight.

### 2.3 Glucosinolates

Glucosinolates are also secondary plant metabolites and popular in the food industry because of their health benefits owing to their antioxidant, antimicrobial, and nutraceutical properties (Förster, Ulrichs, Schreiner, Müller, & Mewis, 2015). Breakdown of glucosinolates releases isothiocyanates due to the activation of the myrosinase enzyme during chewing or food processing. Isothiocyanates were shown to exhibit anticancerous activity (Wu, Zhou, & Xu, 2009). The *M. oleifera* had a higher content of aromatic glucosinolates in the plant parts compared to the other members that grouped under order Brassicales (Bennett et al., 2003). The same authors also stated that young leaves contained approximately 116 mg/g DW, whereas older leaves showed 3 mg/g DW, which showed the glucosinolate content declined with increasing leaf maturity (Bennett et al., 2003).

Glucosinolates, 4‐O‐(a‐L‐rhamnopyranosyloxy)‐benzylglucosinolate (glucomoringin), were identified as a major component of moringa (*M. oleifera* leaves) (Amaglo et al., 2010; Bennett et al., 2003). Moringa variety ICG‐42 of *M. oleifera* was reported to contain approximately 28 μmol/g glucosinolates. Reports of Chen and Zhu (2008) stated that the total glucosinolate content of subsp. *Brassica rapa* chinensis known as pakchoi was 20 mg/100 g FW. Chen and Zhu (2008) also reported six aliphatic glucosinolates (glucoerucin, glucoraphanin, sinigrin, gluconapin, glucobrassicanapin, and progoitrin) and four indolic glucosinolates (4‐hydroxyglucobrassicin, glucobrassicin, 4‐methoxyglucobrassicin, and neoglucobrassicin). However, no detailed reports of the glucosinolate profile in *B. rapa* chinensis grown in *South* Africa are available.

## 3 IMPACT OF GEOGRAPHICAL LOCATION, GENOTYPE, AND AGRONOMY PRACTICES ON DIETARY PHYTOCHEMICALS

Geographical locations, environmental factors, and agronomic practices can affect the composition and the content of phytochemicals in fresh produce (Tiwari & Cummins, 2013). *Amaranthus hybridus* from Vhembe District demonstrated higher (1313 mg/kg DW) total carotenoid content than samples from the Rustenburg District in South Africa (van der Walt, Loots, Ibrahim, & Bezuidenhout, 2009). Similarly, *Cleome gynandra* from Rustenburg District showed higher 1623 mg/kg DW total carotenoid content compared to those from the Capricorn District. Higher, total phenol content was reported in *A. hybridus* (21,812 mg/kg DW) and *C. gynandra* (19,239 mg/kg DW) leaves obtained from Rustenburg District (van der Walt et al. (2009). Ferulic acid concentration in moringa leaves grown in Haiti, Chad, and southwestern Algeria varied from 66.1 mg/kg to 96.9 mg/kg (Leone et al., 2015). The said authors commented that the β carotene content of moringa leaves obtained from Chad and Algeria was similar to the levels found in South African moringa leaves, but the concentrations were lower than that noted in the leaves from India. Also, a comparative study conducted by Ndhlala et al. (2014) clearly demonstrated that the total phenolic and flavonoid content of moringa cultivars grown in different geographical regions contained differ between the locations. Cultivars obtained from Thailand (TOT5169) and South Africa (Silver Hill (SH)) showed higher total phenolic content of approximately 1500 mg/kg based on dry weight. It is also noteworthy that the variation in total phenolic content between the cultivars Silver Hill South Africa and Limpopo. Cultivar Limpopo showed significantly lower total phenolic content than the cv. Silver Hill (Ndhlala et al., 2014). Based on the investigations conducted by Ndhlala et al. (2014), Thailand cultivars TOT4100; TOT4951 and cv. Limpopo from South Africa showed higher flavonoid catechin content around 40 mg/kg. The antioxidant scavenging activity was also noted to vary among the cultivars investigated, and cv. CHM Silver Hill (South Africa) showed the highest activity (32.56 EC50 (μg/ml)) (Ndhlala et al., 2014). However, the correlation between the antioxidant activity and the phytochemical components in moringa cultivars needs to be established.

The phytochemical composition can also vary with a genotype and between different accessions (Kebwaro, 2013). Considering the total phenolic compounds in five spider plant (*C. gynandra*) accessions, it was evident that UGSF accession contained higher total phenol content (14,070 mg/kg) and quercetin (37,090 mg/kg) (Kebwaro, 2013). Furthermore, the maturity stages after plating or the related flowering stage also influenced the phytochemical content in traditional vegetables (Kebwaro, 2013). Rutin content differed among amaranth species at the stage of blossoming (Kalinova & Dadakova, 2009; Martirosyan, 2001). Light quality such as higher UV‐B radiation can also favor the accumulation of rutin and rutin glucosidase in plants (Suzuki, Honda, & Mukasa, 2005). The concentrations of flavonol, quercetin, kaempferol, and isorhamnetin between the varieties of Cowpea leaves varied remarkably (Mduma, 2010). Variety Dakawa contained higher amounts of quercetin (654.8 mg/kg) compared with var. Ex‐Iseke (585.8 mg/kg) (Mduma, 2010). Also the concentrations of kaempferol (224.5 mg/kg) and isorhamnetin (117.8 mg/kg) were higher in var. Ex‐Iseke than in var. Dakawa (Mduma, 2010).

Similarly, moringa cultivars San Pedro and Lombardia contained higher concentrations of chlorogenic acid quercetin content compared to rutin, luteolin, apigenin, and kaempferol (Valdez‐Solana et al., 2015). Based on the findings of Valdez‐Solana et al. (2015), cultivar Lombardia showed an overall higher concentration of chlorogenic acid (479.53 mg/kg), rutin (845.25 mg/kg), and luteolin (94.27 mg/kg), while cultivar San Pedro revealed higher amounts of apigenin (24.41 mg/kg). *Moringa oleifera* is a commonly cultivated moringa species, and *M. ovalifolia* is known as African moringa grown wildly in the southern African region, especially from central to southern Namibia and in southwestern Angola (Ananias, 2015; Dyer, 1975). In Namibia, *M. ovalifolia* leaves obtained from two sites showed a higher total phenolic content (Okaukuejo 168681.6 mg/kg and Tsumeb 151088.5 mg/kg) on a dry weight basis and antioxidant reducing power (Ananias, 2015). According to the reports of Ananias (2015), *M. ovalifolia* contains the three main flavonols, quercetin (11909.31 mg/kg DW), myricetin (581.62 mg/kg, DW), and kaempferol (283.19 mg/kg, DW). Ananias (2015) stated that although the distribution of components of flavonoids is similar to those present in *M. oleifera* leaves, based on the investigations of Pakade et al. (2013), *M. oleifera* contained higher concentrations of quercetin (1362.6 mg/kg DW), kaempferol (1933.7 mg/kg DW), and myricetin (1296.6 mg/kg DW). *Moringa ovalifolia* obtained from the west of Okahandja, Otjozondjupa region, showed a higher content of quercetin (16844.5 mg/kg DW) and kaempferol (590.53 mg/kg DW) (Ananias, 2015). Findings of Ananias (2015) further confirmed that quercetin was the predominant flavonol in the leaves obtained from four different regions or sites in Namibia. The Indian varieties (PKM‐1 and PKM‐2) contain greater content of quercetin and kaempferol (Saini, Sivanesan, & Keum, 2016) than the African indigenous moringa (Coppin, Xu, Chen, & Wu, 2013). It is also noteworthy that the flavone, apigenin, was identified in major moringa cultivars of *M. oleifera* from Pakistan (Saini et al., 2016).

UHPLC‐qTOF‐MS analysis of *M. oleifera* and *M. ovalifolia* leaves revealed 14 flavonoids as aglycones or their glycosides (sugar attachment) [apigenin 6,8 C‐dihexose, kaempferol acetyl dihexose, quercetin acetyl dihexose, quercetin hexose, quercetin hydroxy‐methylglutaroyl hexose, quercetin acetyl hexose, kaempferol hexose, isorhamnetin hexose, quercetin malonyl hexose, kaempferol hydroxy‐methylglutaroyl hexose, kaempferol acetyl hexose, kaempferol malonyl hexose, isorhamnetin hydroxy‐methylglutaroyl hexose, isorhamnetin acetyl hexose] in *M. oleifera* from South Africa, whereas *M. oleifera* from Namibia contains only 10 flavonoids and *M. ovalifolia* showed only three flavonoids (quercetin rutinoside, kaempferol rutinoside, and isorhamnetin rutinoside) (Makita et al., 2017). The genetic factor could be responsible for the observed difference in the flavonoid composition in both species rather than the environmental condition (Makita et al., 2017). However, although flavonoid aglycones are similar, their sugar attachments due to glycosylation varied between the Pakistan varieties of *M. oleifera* Lam (“Tumu,” “Sunyaw,” “Kumasi,” “Techiman,” “China,” “Pakistan Black,” “Pakistan White”) (Nouman et al., 2016) and *M. oleifera* from South Africa (Makita et al., 2017). Also, variety “Pakistan Black” is rich in quercetin‐3‐acetyl‐glucoside 51.98 mg/kg, whereas varieties “Sunyaw” (52.78 mg/kg) and “Kumasi” (53.37 mg/kg) revealed higher amounts of quercetin‐3‐sophoroside on a dry weight basis (Nouman et al., 2016). Varieties “Kumasi” (39.8 mg/kg) and “Pakistan White” (38.86 mg/kg) revealed a higher content of kaempferol‐3‐glucoside (Nouman et al., 2016). Variety “China” and “Pakistan Black” showed remarkably higher amounts of kaempferol‐7‐glucoside (61.99 mg/kg) and apigenin‐7‐ rutinoside (26.44 mg/kg), respectively, than all the other varieties (Nouman et al., 2016). Apigenin‐8‐C‐glucoside was higher in varieties “Tumu” (47.78 mg/kg) and “China” (42.01 mg/kg) (Nouman et al., 2016). Among the hydroxycinnamic acids, 3‐caffeoylquinic acid (chlorogenic acid) was predominant in all seven varieties and “Sunyaw” and “Pakistan White” showed higher contents of 103.30 mg/kg and 100.08 mg/kg, respectively (Nouman et al., 2016). The β‐carotene content in the leaves of different Amaranthus spp, *A. blitum*,* A. hybridus,* and *A. dubius,* also varied from 53 to 68 mg/kg at the vegetative stage, and it declined from 32 to 42 mg/kg at postflowering stage, which indicates the importance of harvesting maturity (Akinyi Nyonje, 2015). In *C. gynandra,* an increase in quercetin content with increasing maturity during flowering after the 6th week of planting (Kebwaro, 2013).

Optimal nitrogen and sulfur status of the plant greatly affect the glucosinolate concentration (Schonhof, Blankenburgh, Műller, & Krumbein, 2007). It has been shown that the plant nitrogen status positively favors the carotenoids and negatively influences the accumulation of total phenols (Chenard, Kopsell, & Kopsell, 2005; Oloyede, Adebooye, & Obuotor, 2014). Cultivation of these wild traditional vegetables with a different source of nitrogen fertilizer application and different source of nitrogen application differently affected the quercetin accumulation in *C. gynandra* at harvest (Kebwaro, 2013). Moreover, the organic farming improves the glucosinolates and phenolics in the plant parts compared to the inorganic farming mainly due to the “stimulation of biotic stress” (Francisco et al., 2016). Also, based on several reports, it is evident that low N application rate favors the accumulation of total phenolic and flavonoid contents in crops (Stefanelli, Goodwin, & Jones, 2010; Zhu, Lin, Jin, Zhang, & Fang, 2009). Francisco et al. (2016), using the hypothesis of Bryant, Chapin, and Klein (1983) in their review, explained that lower availability of N causes lower N uptake by the crop and inhibits the production of N‐based secondary metabolites (e.g., alkaloids) and thereby increases the availability of carbon that can be used for the flavonoid biosynthesis.

Glucosinolate concentrations in the plant parts seem to vary according to the stages of light cycles, and the arid and semiarid environments where water resources are limited can also induce the production of glucosinolates and phenolic compounds (Koyama, Ikeda, Poudel, & Goto‐Yamamoto, 2012). Light quality (Koyama et al., 2012), shade (Agati, Azzarello, Pollastri, & Tattini, 2012), and temperature (Azuma, Yakushiji, Koshita, & Kobayashi, 2012) were also reported to affect the flavonoid contents in crops. The above‐mentioned abiotic factors stimulate the induction of the gene expression and up‐regulation of mRNA involved in several pathways of shikimate during the biosynthesis of secondary metabolites (Petrussa et al., 2013).

## 4 EFFECT OF POSTHARVEST STORAGE AND PROCESSING OPERATION ON PHYTOCHEMICALS IN TRADITIONAL LEAFY VEGETABLES

Recently, Amaranth spp. *Brassica rapa* subsp. *chinensis* have become popular at formal markets and supermarkets as ethnic commodities. Bi‐orientated polypropylene packaging containing 4.3% O_2_ and 7.3% CO_2_ *A. cruentus,* 5.6% O_2_ and 6.7% CO_2_ for *S. reflexum,* and 2% O_2_ and 7% CO_2_ for *B. rapa* subsp. *Chinensis* helped to retain the phytochemicals (total phenols and flavonoids) at the market shelf temperature 10*°*C (Mampholo et al., 2013, 2015). The packaging at the *low* temperature at the market shelf significantly extended the shelf life up to 8 days for *A. cruentus* and up to 10 days for *B. chinensis* and *S. reflexum* (Mampholo et al., 2013, 2015).

It is well known that cooking practices (thermal processes) can affect polyphenols by oxidation in traditional leafy vegetables (Kebwaro, 2013). The changes in phytochemical composition during thermal processing greatly depend on the temperature and duration (time) (Kebwaro, 2013). *Cleome gynandra* leaves boiled for 15 min in water retained 71.42% flavonoids, 37.62% total phenolic compounds, and 51.05% tannin (an antinutritive compound) (Kebwaro, 2013).

Drying is the most common method adopted in food preservation to store traditional leafy vegetables, and sun drying is a popular method used by the rural communities (Mduma, 2010). Exposure of the leafy vegetable to direct sunlight can favor rapid degradation of β‐carotene. Therefore, solar dryers are recommended so that the vegetables can be dried in solar cabinets (Mduma, 2010). Higher levels of carotenoids were lost in amaranthus and cowpea leaves during the open sun‐drying process compared to solar drying (Svanberg, 2007). The direct sun‐drying method was reported to result in 58% loss of β‐carotene in cowpea leaves (Ndawula, Kabasa, & Byaruhanga, 2004). However, solar drying improved the retention β carotene in amaranth, cassava, and pumpkin leaves compared to the amount retained after direct sun drying (Mduma, 2010). The oxidation process of β carotene by the enzymes peroxidase and lipoxygenase (Gökmen, 2010) continues during the drying process, unlike in blanching (Gökmen, 2010; Mduma, 2010). Use of antioxidants such as ascorbic acid could improve the retention of β‐carotene during drying. Also, the solar dryer reduced the drying time by 50% and improved the color, texture, and nutritional properties and could be adopted to preserve the β‐carotene in traditional leafy vegetables (Bala, Mondol, Biswas, Chowdury, & Janjal, 2003; Mduma, 2010). Freeze‐drying results in approximately 30–50% loss of the phenolic content (Shofian, Hamid, & Osman, 2011), while microwave vacuum drying reduces the loss of phenolic compounds (Mejia‐Meza, Yanez, & Remsberg, 2010). *Moringa oleifera* leaves dried using a heat pump‐assisted dehumidified air dryer at 50°C for 55 min with air velocity at 0.5 m/s and thereafter vacuum packed PET/Al/PE (high‐density polyethylene and metallized films) and stored at 15°C showed an increase in quercetin content from 796.9 to 1395.9 mg/kg (Potisate, Kerr, & Phoungchandang, 2015). The PET/Al/PE reduced the amount of “moisture pickup for a product in this package” and the hydrolysis of phenolic compounds or removal of sugar attachments from quercetin during storage favored the increase in quercetin during storage (Potisate et al., 2015).

Blanching is a beneficial process in food processing that inactivates enzymes (peroxidase and lipoxygenase) that are involved in carotenoid destruction through oxidation (Mduma, 2010). Blanching enabled the retention of 15% of β‐carotene in cowpea leaves (Ndawula et al., 2004). The canning process involves a higher temperature (100 ^◦^C) and longer duration (1 h), and it was reported to convert 75% of trans‐β‐carotene to 13 or 9 isomers that are not absorbed by the body (Van het Hof, Gärtner, West, & Tijburg, 1998). The aforesaid authors indicated that mild heating such as steaming was demonstrated to improve the extractability of β‐carotene from vegetables as well as its bioavailability. Dehydration of leafy vegetables also favors the loss of β‐carotene mainly by increasing the surface area (Mduma, 2010). Extensive cutting or chopping or grinding also favors the destruction of β‐carotene and polyphenols in vegetables (Abid, Jabbar, & Wu, 2013), therefore blanching prior to the cutting or chopping process (Bohn, 2014).

Using sunflower oil or red palm oil during cooking improved the accessibility of β‐carotene content by 39–94% of traditional vegetables such as amaranth, sweet potato, pumpkin, and cassava leaves (Hedren, Mulokozi, & Svanberg, 2002). It was also reported that generally leaves are subjected to a drying process prior to cooking and β‐carotene decreased to 19.4% during drying (Ndawula et al., 2004). However, Medoua and Oldewage‐Theron (2014) found that a 25‐min cooking process with water to just cover the leaves decreased the total polyphenols to 58.33%, myricetin to 51.97%, and kaempferol to 40.86%, while among the three flavonols, quercetin was least affected (25.44% reduction). In order to improve the intake of moringa, Kiranawati and Nurjanah (2014) had developed moringa noodles. The sautéing cooked (cooking quickly in a minimal amount of fat over relatively high heat) moringa noodles improved the milk production in rats (Kiranawati & Nurjanah, 2014).

## 5 BIOAVAILABILITY OF DIETARY PHYTOCHEMICALS

The beneficial effect of dietary phytochemicals depends on their bioavailability (absorption, distribution, metabolism, and excretion) which is mainly dependent on the structure of the phytochemical and food matrix. Furthermore, the term bioavailability can be defined according to Thilakarathna and Rupasinghe (2013) as the rate of absorption and the availability at the site of action is very important for a bioactive compound to be effective within biological systems and thus be “bioavailable.” Based on this explanation, it is clear that the concentration of the compound and its metabolites at the site of action is more important than the concentration of a dietary phenolic compound in a particular food. Scalbert and Williamson (2000) and Thilakarathna and Rupasinghe (2013), in their reviews, reported that factors such as “class of phenolic compounds, complex structures of phenolic compounds, degree of polymerization and molecular weights, glycosylation, metabolic conversion process and interaction with colonic microflora” affect the bioavailability of the dietary phenolic compounds. As mentioned, recent research has focused on the impact of dietary polyphenols on the gut microbiota composition and the effect of gut microbiota on the biotransformation of phenolic compounds, their bioavailability, and human health (Ozdal et al., 2016). Flavanones showed higher bioavailability than flavonols and flavan‐3‐ols mainly due to the lesser degradation by the gut microflora and the greater bio‐accessibility for intestinal absorption (Ozdal et al., 2016). Furthermore, the bioavailability of catechins (tea) was improved by supplementation with steamed rice. Higher amounts of proline‐rich proteins in the rice endosperm bind with the epigallocatechingallate and epicatechin gallate and convert them to nongallated catechins in the small intestines (Monobe, Ema, Tokuda, & Maeda‐Yamamoto, 2011). The authors also mentioned in their research findings that the nongallated catechins are more readily absorbed than the gallated catechins. In some cases, the heating process can break the plant cell walls and thereby mediate the release of polyphenols during digestion (Bohn, 2014). Cutting and grinding of blanched vegetables can increase the bio‐accessibility of polyphenols by increasing the surface area for the activity of the digestive enzymes (Abid et al., 2013). Bio‐accessibility can be defined as the fraction of a compound that is available for the absorption by the gut (Alminger et al., 2014). Domestic cooking influences the bioavailability of naringenin, and chlorogenic acid increased amounts of theses phenolic compounds in human blood plasma compared to the consumption of fresh cherry tomatoes (Bugianesi, Salucci, Leonardi, & Maiani, 2005). However, higher temperature and processing time can negatively affect the naringenin and chlorogenic acid concentration in the vegetables (Bugianesi et al., 2005).

Bioavailability of quercetin is affected by the differences in its conjugated glycosides, but higher bioavailability of quercetin can be obtained from quercetin glucoside than quercetin rhamnoside and quercetin galactoside (Kasikci & Bagdatlioglu, 2016). Furthermore, quercetin bioavailability can be improved when quercetin is consumed as a cereal bar ingredient instead of a capsule (Egert et al., 2012). The authors explained that the manufacturing process helps to improve the homogeneous solid dispersion of quercetin in the presence of the ingredients of a cereal bar. Also, the presence of dietary fat (Kasikci & Bagdatlioglu, 2016; Lesser, Cermak, & Wolffram, 2004) and fructooligosaccharide (Matsukawa et al., 2009) in the food matrix was demonstrated to improve the bioavailability of quercetin. The novel nano‐encapsulation technology (Hu, Liu, Zhang, & Zeng, 2017), on the one hand, has been demonstrated to increase the bioavailability and to improve the interaction of polyphenols with the food matrix during digestion, especially by improving their solubility. On the other hand, bioavailability of β‐carotenes can be improved by a food processing method and pureed; thermally processed spinach was shown to influence the blood plasma response of β‐carotene (Palafox‐Carlos, Ayala‐Zavala, & Gonz′alez‐Aguilar, 2011; Rock & Swendseid, 1992). The role of dietary fiber in the bio‐accessibility and bioavailability of phytochemicals of fruit and vegetables indicates that soluble dietary fiber in the gut could inhibit the absorption of carotenoids as lipid soluble compounds (Palafox‐Carlos et al., 2011).

During freezing of *B. rapa* subsp. chinensis, typical blanching protocols are recommended prior to freezing in order to inactivate the myrosinase enzyme and thereby reducing the bioavailability of isothiocyanates. Myrosinase is heat sensitive (thermolabile) and denatures at 60 C for 10 min (Francisco et al., 2016; Van Eylen, Oey, Hendrickx, & Van Loey, 2007). Furthermore, food preparation methods such as chopping and grinding can affect the glucosinolate content due to the tissue damage‐induced myrosinase activity that results in the production of isothiocyanates (Francisco et al., 2016). Steaming for 15 min was shown to retain the glucosinolates to a great extent in brassica vegetables (Francisco et al., 2010).

## 6 DEVELOPMENT OF FOOD PRODUCTS WITH TRADITIONAL LEAFY VEGETABLES FOR DIETARY DIVERSIFICATION

Nestlé included morogo (Amaranth) leaf to flavor their new line of Maggi two‐minute noodles (Greve, 2015). Moringa dried leaf was used to fortify the noodles to improve the iron and dietary phytochemicals. Similarly, “moringa fortification was adopted to increase the nutrient level in children and incorporation of 20% moringa powder in cocoa powder” (Gopalakrishnan, Doriya, & Santhosh Kumar, 2016). Different percentages of moringa leaf powder tested in the chocolate fortification indicated that 20% moringa incorporation in cocoa powder was ideal (Gopalakrishnan et al., 2016). The addition of moringa powder to cocoa during chocolate production can help to reduce childhood obesity (Morsy, Mohamed Rayan, & Youssef, 2015). The said authors also demonstrated the production of rice extrudate products containing 2% Jew's mallow (*C. olitorius* L. Leaves) dried leaves for healthy snack food production. Production of health beverages including moringa leaf as an ingredient with beetroot leaves was also reported (Vanajakshi, Vijayendra, Varadaraj, Venkateswaran, & Agrawal, 2015). The authors demonstrated the fermented moringa leaves in a beetroot‐based beverage containing 1:2 moringa leaf paste and beetroot showed 20.79% radical scavenging activity with a phenolic content of 5 mg/ml and 30‐day shelf life at 4°C with a good viable lactic population at 6.5 pH.

## 7 ANTINUTRITIVE COMPOUNDS IN TRADITIONAL LEAFY VEGETABLES

It is clearly evident, based on several research reports, that the traditional leafy vegetables contain non‐nutrient or antinutrient bioactive phytochemicals. Some of these phytochemicals were found to pose some toxicity when consumed in large quantities or over a long period (Smith & Eyzaguirre, 2005).

Oxalate and oxalic acid are organic acids known as antinutrient compounds present in various traditional vegetables. Levels of oxalate around 780 mg/kg according to dry weight in *Solanum nigrum* L. Var. *virginicum* leaves are higher than other antinutrients such as saponins (2.5 mg/kg DW), tannins (1.9 mg/kg DW), phytic acid (8.2 mg/kg DW), or cyanide (106.3 mg/kg DW) (Akubugwo, Obasi, & Ginika, 2007). A high oxalate content is reported in cowpea leaves var. Dakawa (4180 mg/kg) and Ex‐Iseke (3480 mg/kg) (Mduma, 2010). Molina et al. (2016) reported a high content of oxalates (216.27 to 368.5 mg/kg) and phytates (2090 to 2500 mg/kg) in *A. dubiu*. The concentration of oxalates and phytates in *A. dubius* leaves varied according to the harvesting season, especially during the dry seasons when the oxalates and phytates increased up to 368.5 mg/kg and 2500 mg/kg (Molina et al., 2016).

The environmental conditions favored the stress‐related changes and have stimulated the synthesis of oxalates and phytates in the plant (Bohnert, Nelson, & Jensen, 1995; Molina et al., 2016) oxalates interfere with the absorption of Ca^2+^, Mg^2+^, and Fe^2+^ (divalent ions) (Reddy, Sathe, & Salunkhe, 1982). Oxalic acid can form insoluble iron–oxalate complexes that render the iron unavailable and inhibit their intestinal absorption (Kawazu, Okimura, Ishii, & Yui, 2003). Findings of Leone et al. (2015) showed that antinutritive component phytates in moringa leaves obtained from Haiti, Chad, and southwestern Algeria varied from 29.5 to 30.3 mg/kg DW. The phytates in moringa leaves from these regions were greater than those found in legumes and cereals (Leone et al., 2015; Reddy, 2002), but slightly lower compared to the amounts found in wheat bran, as reported by Fardet (2010). Phytate (phytic acid) chelates metal ions (nonheme iron), especially iron and zinc, and inhibit or make them unavailable for intestinal absorption (Gibson, Perlas, & Hotz, 2006; Zijp, Korver, & Tijburg, 2000). The inhibitory effect of phytic acid can be prevented by the intake of vitamin A and β‐carotene (Layrisse et al., 2000), and ascorbic acid (Davidson, 2003; Teucher, Olivares, & Cori, 2015)‐rich food at appropriate concentrations can favor the nonheme iron absorption. Vitamin A or β‐carotene favors iron absorption by forming soluble iron complexes (Layrisse et al., 2000). Ascorbic acid favors the optimal acidic conditions, especially in the stomach and intestines for the absorption of iron and also acts as a chelating agent of ferric iron so that it is stable and in a soluble form, even at a higher pH (Bohn et al., 2008; Teucher et al., 2015). Also, ascorbic acid prevents the reduction in ferric iron to ferrous and the precipitation of ferric hydroxide (Bohn et al., 2008; Teucher et al., 2015). Conventional cooking of vegetables can reduce the phytic acid to some extent; however, methods such as soaking in an acid medium, lactic acid fermentation, and sprouting can help to reduce the phytic acid concentration (Bohn et al., 2008). Use of phytase enzymes can be recommended during food processing to reduce the phytate content; however, production of thermally stable phytase needs to be cost‐effective and an efficient process for the food industry (Bohn et al., 2008).

Tannin is a group of phenolic compounds responsible for the astringent (bitter mouthfeel) sensory attribute (Egbuna & Chieneye, 2015). Tannin binds and precipitates proteins, amino acids, and alkaloids and thereby reduces their bioavailability (Egbuna & Chieneye, 2015). Tannin content in Amaranthus spp is higher around 157 mg/kg, but in *A*. *cruentus* it is around 7.6 mg/kg, which explains the preferred traditional food preference of *A*. *cruentus* by the consumers. Tannin content in *C. gynandr*a (spider plant) from Zimbabwe, on the one hand, was reported to contain 190 mg/kg, and it is comparable to the amount found in commonly consumed lettuce (Chipurura, 2010). On the other hand, the different genotypes of *C. gynandr*a, such as CGSKGP (Seke District, Zimbabwe), CGMRR (Marondera, Zimbabwe), and CGSKP (Seke District, Zimbabwe), had lower tannin content than the amounts (Chipurura, 2010). The tannin content between these genotypes varied from 230 to 490 mg/kg (Chipurura, 2010). However, *A. hybridus* contains 0.60 mg/kg oxalate, 4.12 mg/kg phytate, and 0.20–0.19 mg/kg tannin (Agbaire, 2012). Some reports sates tannin or saponin were not detected in Amaranthus spp (Uusiku et al., 2010;). The difference in non‐nutritive components can also vary according to geographical location, soil nutritional components, and abiotic stress experienced by the plants. Common antinutritive compounds are summarized in Table 4.

**Table 4:** Common antinutritive compounds in traditional (African) vegetables

Traditional leafy vegetable	Antinutritive compounds	References
*Solanum nigrum*		
* S. nigrum* L. Var. *virginicum*	Oxalate content	
	780 mg/kg DW	Akubugwo et al. (2007)
	Saponins	
	2.5 mg/kg DW	Akubugwo et al. (2007)
	Tannins	
	1.9 mg/kg DW	Akubugwo et al. (2007)
	Phytic acid	
	8.2 mg/kg DW	Akubugwo et al. (2007)
	Cyanide	
	106.3 mg/kg DW	Akubugwo et al. (2007)
*Vigna unguiculata*		
* V. unguiculata* var. Dakawa	Oxalate content	
	4180 mg/kg DW	Mduma (2010)
* V. unguiculata* var Ex‐Iseke	3480 mg/kg DW	Mduma (2010)
Amaranthus spp		
* A. dubiu*.	216.27–368.5 mg/kg DW	Molina et al. (2016)
* A. hybridus*	0.60 mg/kg DW	Agbaire (2012)
	Phytates	
	2090 to 2500 mg/kg DW	Molina et al. (2016)
* A. hybridus*	4.12 mg/kg DW	Chipurura (2010).
	Tannin content	
* A*. cruentus	7.6 mg/kg DW	Chipurura (2010)
* A*. cruentus	0.20–0.19 mg/kg DW	Chipurura (2010)
*Spider flower*		
* C. gynandr*a	190 mg/kg DW	Chipurura (2010)

DW, dry weight.

## 8 CONCLUSION

The review reemphasizes that the inclusion of traditional vegetables in a regular diet could provide many health benefits. However, it is evident that there is a significant knowledge gap in the quantification of dietary phytochemicals, especially in commonly consumed traditional vegetables in the Southern African regions. Investigation of phytochemical profiles and their quantities is vital for the establishment of dietary guidelines. Also, it is important to understand the antinutritive components in commonly consumed traditional vegetables. The development of a database on antinutritive components and the contents could help to advance methods to remove or reduce the antinutritive components during food processing and by manipulating agronomy practices. Also, tremendous potential exists for domesticating wild types of vegetables and enhancing their yield through establishing proper agronomic practices. Further research is required to understand the effect of abiotic stress factors on the improvement of dietary phytochemicals. The effects of applications of irrigation, nitrogen, phosphorous and potassium application and organic amendments on yield and concentration of phytochemicals need to be investigated. Also, selection of genotypes or accessions that are rich in beneficial dietary phytochemicals, but low in antinutrients, are important to promote the marketing of traditional vegetables.

Generally, traditional vegetables are sold at the urban markets; therefore, the highly perishable nature of these vegetables affects their marketing. Therefore, adapting appropriate postharvest handling methodologies and the use of appropriate packaging are recommended to reduce the postharvest loss of freshly harvested traditional leafy vegetables in order to retain the dietary phytochemicals at the retailer's shelf during marketing. As boiling was recommended for the retention of phytochemicals and for the removal of antinutritive compounds, the vegetables need to be marketed in fresh form. It is also important to identify the harvestable maturity, cost‐effective methods to enhance shelf life, and appropriate packaging for the traditional leafy vegetables. When developing new value‐added processes for functional food products, it is also important to assess the loss of dietary phytochemicals.

Overall, it is important to promote the consumption of traditional leafy vegetables that possess a wide variety of health‐promoting phytochemicals. Tremendous potential exists to establish improved crop production and food manufacturing practices to optimize the phytochemicals and introduce vegetable‐based functional foods that could combat diseases and obesity while sustaining food security in the southern African region.

## CONFLICT OF INTEREST

The authors declare that they have no conflict of interest.
